# Neurophysiological Responses to Inhalation of *Osmanthus fragrans* Volatiles: A Combined Electronic Nose and Electroencephalogram (EEG) Study on Concentration-Dependent Effects

**DOI:** 10.3390/plants15132006

**Published:** 2026-06-29

**Authors:** Seong Jun Hong, Hyeonjin Park, Younglan Ban, Se Young Yu, Hee Sung Moon, Ji Sun Kim, Daeyong Shin, Kiseong Kim, Young Jun Kim, Jae Kyeom Kim, Eui-Cheol Shin

**Affiliations:** 1 Department of GreenBio Science (BK21)/Food Science and Technology, Gyeongsang National University, Jinju 52828, Republic of Korea; sjhong@kmu.ac.kr (S.J.H.);; 2 Department of Food Science and Technology, Keimyung University, Daegu 42601, Republic of Korea; 3 Graduate School of Information Communications, Hanbat National University, Daejeon 34158, Republic of Korea; 4 Graduate School of Design, Kookmin University, Seoul 02707, Republic of Korea; 5 Department of Food and Biotechnology, Korea University, Sejong 30019, Republic of Korea

**Keywords:** *Osmanthus fragrans* var. *aurantiacus*, E-nose, EEG, sex-specific differences, sLORETA

## Abstract

*Fragrant olive* (*Osmanthus fragrans* var. *aurantiacus* (*O. fragrans*)) extract is known to influence neurophysiological responses through inhalation, yet research on concentration-dependent effects and sex-specific variations remains insufficient. This study utilized an electronic nose (E-nose), electroencephalography (EEG), and standardized low-resolution electromagnetic tomography (sLORETA) to characterize the volatile profiles and neurophysiological impacts of *O. fragrans* at 3% and 5% concentrations. E-nose analysis identified 48 volatile compounds, with chemometric modeling (PCA, HCA) showing clear discrimination between concentrations. EEG results demonstrated that inhalation induced significant concentration-dependent changes—specifically increasing sedation-related alpha waves and decreasing tension-related gamma waves—with 5% *O. fragrans* eliciting more widespread cortical responses than the 3% concentration. Notably, no significant sex-related differences were observed in general EEG patterns; however, sLORETA revealed that 5% inhalation specifically suppressed high beta and gamma activities in male participants within Brodmann areas 13, 21, 22, and 44, regions associated with emotional and multisensory processing. In conclusion, this study successfully quantified the relationship between volatile profiles and human brain responses using an integrated biomimetic and neurophysiological approach. These findings provide objective evidence that *O. fragrans* inhalation, particularly at 5%, modulates neural oscillations toward a relaxed state, offering valuable data for olfactory perception and potential applications as functional volatile compounds.

## 1. Introduction

*O. fragrans* is one of the representative ornamental plants and flowers, and this plant is mostly distributed in Asia countries, including China, India, Japan, Thailand, and the southern area of Korea. Furthermore, *O. fragrans* is known as the sweet olive, and the flower of this plant is generally used for natural food, anti-browning, and anti-oxidant flavor additives for food preservation [1]. The edible flower is used in various food ingredients for garnishing and flavor, improving the flavor and color characteristics. To date, the utility of edible flowers has increased due to various physiological functions [2]. Recently, *O. fragrans* extract has some neuroprotective effects, including a reduction in lipid peroxidation in cortical neurons, increased sedation, and decreased appetite by reducing mRNA expression of orexigenic neuropeptides. Inhalation of volatiles can stimulate the olfactory and autonomic nervous system (ANS), and ANS mainly regulates the balance between sympathetic and para-sympathetic nerves [1,3]. Additionally, inhalation of *O. fragrans* oil reduced anxiety in anxious patients undergoing colonoscopy [1,3,4]. Olfactory stimuli generally play a key role in assessing foods and natural products. Accordingly, different volatile compound profiles in foods and natural products can affect the sensory quality and recognition [5].

The process of inhaling volatile compounds and stimulating the olfactory system has been reported [6]. The binding of these odorous molecules to olfactory receptors initiates and transmits olfactory signals to areas of the brain responsible for autonomic functions. It has been established that the olfactory stimulation caused by these volatile compounds can have physiological effects by affecting the ANS. Different types of volatile compounds can induce various neurophysiological effects, and the concentration of volatile compounds is also a significant factor that influences these effects [1,5,6,7]. Olfactory receptors play a key role in the cognition of odors by binding with volatile compounds. Furthermore, olfactory receptors are also responsible for the regulation of various physiological effects, including the regulation of hepatic steatosis, adiposity, blood pressure levels, and wound healing [8,9]. The concentration of volatile compound molecules is important for the composition of the combinatorial receptor coding. When the concentration is low, the primary olfactory receptors of each odor molecule are stimulated. However, a large number of secondary olfactory receptors are also stimulated at a high concentration, which also causes changes in the combination code. This concentration-dependent variation alters the spectrum of activated olfactory receptors, modifying the combinatorial receptor coding transmitted to the brain and consequently shifting the human neurophysiological response [7,10,11,12,13]. Furthermore, high doses of volatiles inhalation can induce side effects. Previous studies reported that low doses of patchouli and basil essential oils inhalation induced anti-obesity effects. However, the inhalation of high doses of these essential oils induced side effects, including increased visceral fat mass [5,8]. Olfactory sensory measurement is a relevant tool in discerning food authenticity and guiding food production. This measurement usually relies on human olfactory senses and biomimetic sensory-based machine perception techniques, including E-nose [2]. Human sensory measurement (manual evaluation; panel test) has some limitations, which include relying on linguistic descriptions (subjective) and lack of uniform measurement criteria. In contrast, the measurement of machine perception techniques, such as E-nose, has some advantages, including objectivity, high reproducibility in aroma measurement, and fast detection. Therefore, the application of E-nose has increased, and it is widely used to measure food inspection and quality control. Although the E-nose is a useful tool for measuring food odor characteristics, the machine perception technique lacks emotional understanding, consumer preferences, physiological data, and neurocognitive data [14]. Accordingly, the combined brain response with E-nose is necessary for understanding olfactory characteristics with neurocognitive and volatile compound information.

EEG is a technique that records electrical signals generated and transmitted by stimulated neurons in the brain [15]. EEG is used to assess mental functions and detect human health conditions. Numerous studies have been conducted on EEG observations related to human senses, including responses to odor and taste [15,16,17,18,19,20]. EEG captures amplified intracellular electrical signals from the cerebral cortex via a non-invasive method involving an intact scalp [15]. This method allows for the identification of specific active brain regions at any given time based on brain wave patterns [21]. The simplicity and non-invasiveness of this brain wave measurement make this technique a suitable tool for evaluating the olfactory system when inhaling specific essential oils [15,21]. Brain waves are classified as five main bands: delta (0–4 Hz), theta (4–8 Hz), alpha (8–13 Hz), beta (13–30 Hz), and gamma (>30 Hz), which are naturally exhibited during both resting and active states [22]. Recent research indicates that EEG can detect alterations in an individual’s mental state during and/or following the inhalation of essential oils and volatile compounds [15,22,23,24,25,26].

Recently, standardized low-resolution electromagnetic tomography (sLORETA) has been widely used to investigate brain-localized sources. In general, EEG only provides 2-dimensional (2D) results (scalp electric signals; topography); however, sLORETA can provide 3D results (intracerebral signals; tomography). sLORETA approach can explore the localized signal sources in the brain through the inverse problem based on the Montreal Neurological Institute (MNI) 152 brain standard [22]. Consequently, the exploration of localized sources can detect the Brodmann area(s) through 5000 random trials (permutation). Brodmann area(s), which is divided into a total of 52 areas, is responsible for individual neurophysiological function(s) [22,24].

To systematically evaluate these aroma characteristics, biomimetic sensory machine perception via an electronic nose (E-nose) has been widely adopted for its high reproducibility, objectivity, and rapid volatile profiling [9,10]. While the E-nose system at generating chemical fingerprints and identifying concentration-dependent volatile patterns, such mechanical analysis of volatiles cannot explain or predict the complex, real-time physiological, emotional, and neurocognitive responses triggered in the human brain. Therefore, bridging chemical profiling with human sensory experience necessitates an integrated approach that combines machine perception with objective neuroimaging techniques. Electroencephalography (EEG) and standardized low-resolution electromagnetic tomography (sLORETA) serve as powerful tools to address this limitation; EEG records real-time electrical signals across specific cortical bands, while sLORETA models three-dimensional intracerebral signal sources to pinpoint localized activation within specific Brodmann areas [11,12]. By implementing a combined E-nose and EEG/sLORETA framework, this study comprehensively investigated the concentration-dependent and sex-specific neurophysiological impacts of *O. fragrans* extracts, thereby establishing the scientific necessity and novelty of this research.

## 2. Results

### 2.1. E-Nose and Chemometric Approaches

**Figure 1:**
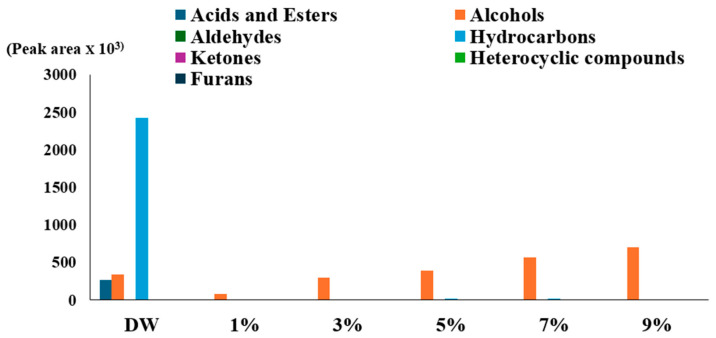
Major volatile compound profiles in different concentrations of *Osmanthus fragrans* extracts using E-nose.

**Figure 2:**
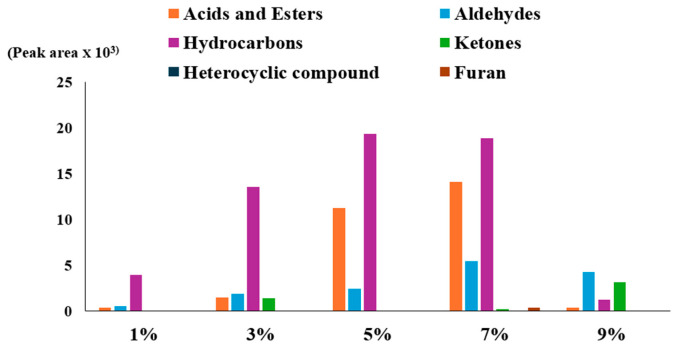
Minor volatile compound profiles in different concentrations of *Osmanthus fragrans* extracts using E-nose.

**Figure 3:**
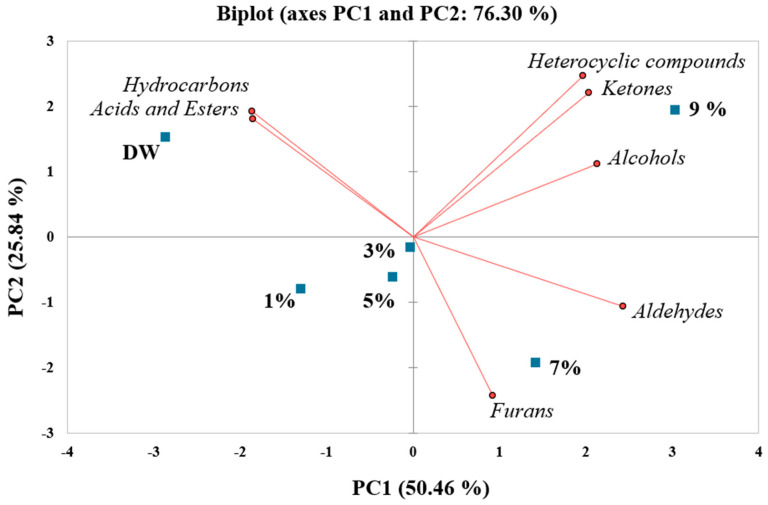
PCA-biplot results (PC1 and PC2 axes) based on the E-nose data.

**Figure 4:**
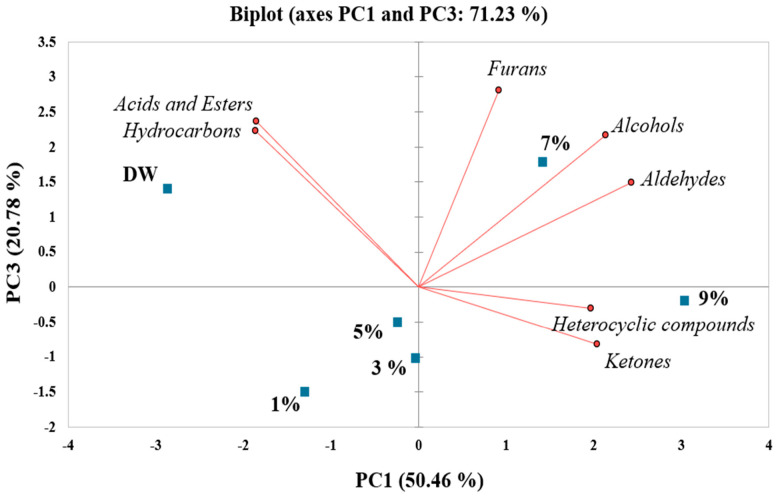
PCA-biplot results (PC1 and PC3 axes) based on the E-nose data.

**Figure 5:**
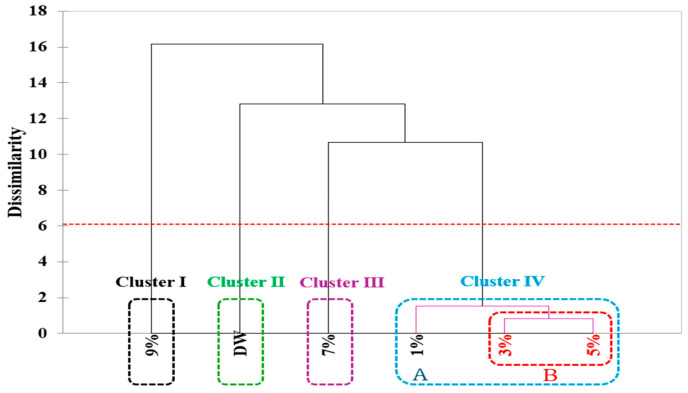
Hierarchical cluster analysis based on E-nose data. The red dotted line represents simialr clustering.

Volatile compound profiles in distilled water (DW) and *O. fragrans* extracts were measured using an E-nose system, and the results are shown in Figure 1 and Table 1. A total of 49 volatiles were identified, which include nine acids and esters, thirteen alcohols, four aldehydes, seventeen hydrocarbons, four ketones, one heterocyclic compound, and one furan. Among these volatile compounds, the number of volatiles was higher in hydrocarbons (17), alcohols (12), and acids and esters (nine). Hydrocarbons were the dominant compounds in the DW. However, alcohols were the dominant compounds in *O. fragrans* extracts, and these volatiles were increased in a concentration-dependent manner. Volatile compound patterns in DW and *O. fragrans* were identified via chemometric approaches (PCA and HCA) based on E-nose data. Several *O. fragrans* concentrations showed different locations, and the results are shown in Figure 2. In PCA results (PC1 and PC2 axes biplot) (Figure 3), the total PCs showed 76.30% variance, including 50.46% (PC1) and 25.84% (PC2). Additionally, 7% and 9% concentration extracts were located on the positive axis of PC1; however, DW, 1%, 3%, and 5% extracts were located on the negative axis of PC1. Unlike the PC1 axis, 7% and 9% extracts were located on the positive axis of PC2; however, 1%, 3%, 5%, and 7% extracts were located on the negative axis of PC2. In the PC1 and PC3 axes biplot (Figure 4), the total PCs showed 71.24% variance, including 50.46% (PC1) and 20.78% (PC3). Additionally, 7% and 9% extracts were located on the positive axis of PC1; however, DW, 1%, 3%, and 5% extracts were located on the negative axis of PC1. Unlike the PC1 axis, DW and 7% extract were located on the positive axis of PC3; however, 1%, 3%, 5%, and 9% extracts were located on the negative axis of PC3. In HCA results (Figure 5) based on PCA data, a 9% extract was classified as cluster I, and DW was classified as cluster II. A 7% extract was classified as cluster III, and 1%, 3%, and 5% extracts were classified as cluster IV. Additionally, cluster IV was separated by sub-clusters A and B. Among all clusters, the significant differences only showed among clusters I-IV; however, subclusters A and B did not show any significant difference(s).

### 2.2. EEG Measurements

**Figure 6:**
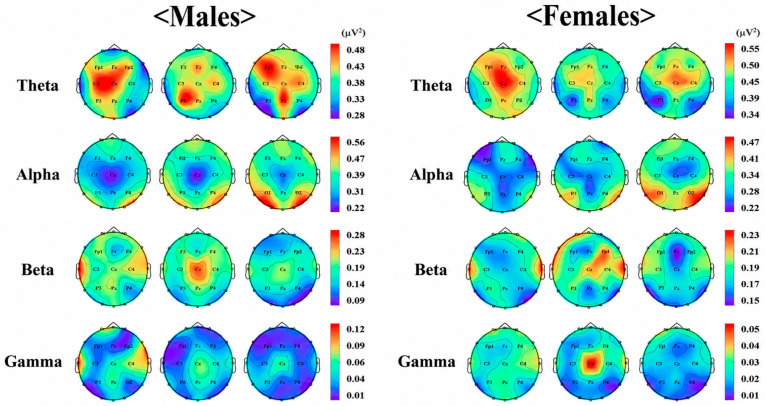
Change in EEG signals by inhalation of 3% and 5% concentrations of *Osmanthus fragrans* extracts in relative theta to gamma waves of males and females.

**Figure 7:**
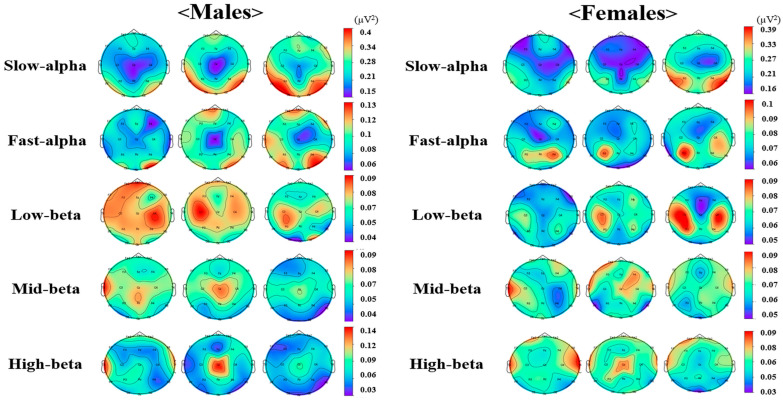
Change in EEG signals by inhalation of 3% and 5% concentrations of *Osmanthus fragrans* extracts relative slow-alpha to high beta waves of males and females.

Effects of *O. fragrans* inhalation on human brain activities were measured using EEG equipment, and the results (RT-RHB) are shown in Figure 6 and Figure 7 (males and females). Significant differences in EEG signals were expressed as box plots, and these results are shown in Figure S1. In male EEG results (Figure 6), RA, RSA, and RFA waves showed an increased tendency after inhalation of *O. fragrans* extracts; however, RB, RG, RLB, RMB, and RHB waves showed a decreased tendency after inhalation of *O. fragrans* extracts. Among all EEG waves, RA, RB, RG, RLB, and RHB waves only showed significant differences before and after inhalation (*p* < 0.05). Among these brain waves, the number of RG electrodes (five sites) showed higher changes compared with other EEG waves. However, RSA, RFA, and RMB waves did not show any significant differences between before and after inhalation states. Among significant differences in EEG waves (Figure S1), RB, RG, RLB, and RHB waves showed significant decreases in some electrodes (*p* < 0.05). However, RA waves showed significant increases in two electrodes (P3 and O1) between before and after inhalation of 5% *O. fragrans* (*p* < 0.05). In significantly decreased EEG waves, RB waves only significantly decreased in the O2 site after inhalation of the 5% *O. fragrans* extract (*p* < 0.05), and RLB and RHB waves significantly decreased in three (P4, O1, and Oz) sites and one (T3) site after inhalation of the 5% *O. fragrans* extract, respectively (*p* < 0.05). In RG waves, the five sites (F7, F8, T3, Pz, and T6) showed a significant decrease after inhalation of the 5% extract (*p* < 0.05), and only a site (T3) showed a significant decrease after inhalation of the 3% *O. fragrans* extract *(p* < 0.05). In RA waves, two sites (P3 and O1) significantly increased after inhalation of the 5% *O. fragrans* extract (*p* < 0.05). Furthermore, unlike the results of E-nose measurement, the results of EEG signals have discriminated according to different concentrations (3% and 5% *O. fragrans*).

In female EEG results (Figure 7), most of the EEG activities showed different imaging patterns according to the inhalation concentrations compared with male results. Among all EEG waves, RA, RG, RSA, RLB, and RHB only showed significant differences before and after inhalation (*p* < 0.05). In RA and RSA waves, five sites (F7, F3, F8, Pz, and P4) significantly increased after inhalation of the 5% *O. fragrans* extract (*p* < 0.05), and seven sites (Fp1, F7, F3, F8, Pz, P4, and O2) significantly increased in RSA waves after inhalation of the 5% *O. fragrans* extract (*p* < 0.05). In RB, RLB, and RHB waves, two sites (Fp1 and F8) of RLB, and two sites (Cz and Oz) of RHB significantly decreased after inhalation of the 5% *O. fragrans* extract (*p* < 0.05). In RG waves, four electrodes (P4, T6, Oz, and O2) significantly decreased after inhalation of the 5% *O. fragrans* extract (*p* < 0.05).

### 2.3. sLORETA Approaches

**Figure 8:**
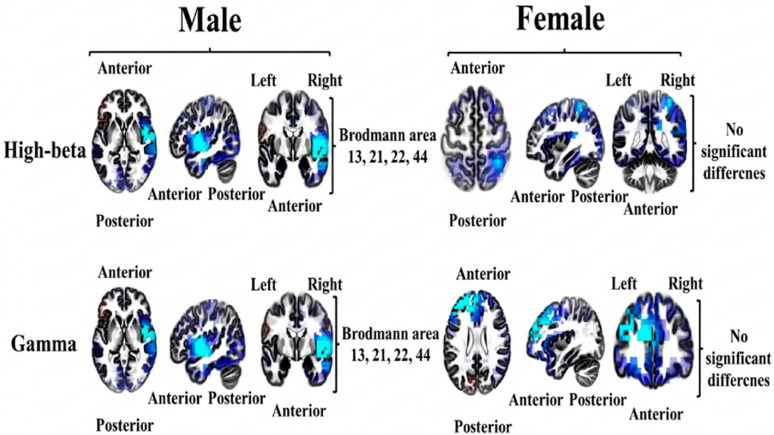
Changes in intracerebral signals by 5% concentration of *Osmanthus fragrans* extract in relative high beta and gamma waves of males and females.

The effects of differential concentrations of *O. fragrans* extracts according to different sex on the intracerebral signals (localized sources) were identified through the sLORETA based on the EEG signals, and these results are shown in Table 2 and Figure 8. Among nine EEG waves, the significant differences were only identified in RHB and RG waves after inhalation of *O. fragrans* extracts through 5000 random trials (permutation) using sLORETA. In RHB waves, the insula (Brodmann areas 13) in the sub-lobar and middle temporal gyrus (Brodmann area 22) in the temporal lobe were significantly decreased in male participants after inhalation of 5% *O. fragrans* extracts (*p* < 0.01). The inferior frontal gyrus (Brodmann area 44) in the frontal lobe and middle temporal gyrus (Brodmann area 21) in the temporal lobe were significantly decreased in male participants after inhalation of 5% *O. fragrans* extracts (*p* < 0.05). However, Brodmann areas did not show any significant changes in female participants after inhalation of 5% *O. fragrans* extracts, except for inhalation concentrations. In RG waves, the insula (Brodmann area 13) in the sub-lobar and middle temporal gyrus (Brodmann area 22) in the temporal lobe were significantly decreased in male participants after inhalation of 5% *O. fragrans* extracts (*p* < 0.01). The inferior frontal gyrus (Brodmann area 44) in the frontal lobe and middle temporal gyrus (Brodmann area 21) were significantly decreased in male participants after inhalation of 5% *O. fragrans* extracts (*p* < 0.05). Consistency with RHB results, RG also did not show any significant changes in female participants after inhalation of 5% *O. fragrans* extracts.

## 3. Discussion

The E-nose system is representative of the chemosensory device, and it is made based on mimicking human olfaction perception and characteristics. E-nose has some advantages, including low cost, fast detection, and objective, and this device is used to show similar results and patterns of human sensory analysis. Additionally, the operation of E-nose is not necessary for the training process compared with human sensory analysis [1,25,26]. In this study, volatile compound profiles in DW and *O. fragrans* extract were identified. Particularly, the major volatiles in *O. fragrans* extracts were identified as alcohols. Chemometric analysis is a mathematical, logical, and statistical method to efficiently manage and interpret chemically derived data [27]. Among the chemometric approaches, PCA and HCA represent the patterns and relationships (similarity or dissimilarity) among samples through the variables [26]. PCA is a valuable statistical tool for compressing large data sets into main variables (summarized variables), which can be represented in simplified and visual pattern data (loading plot and the location of samples). HCA is a useful statistical tool for showing relationships as clusters among samples [26]. Previous studies reported that classifications were represented by PCA and HCA, and chemometric approaches based on the E-nose data may be similar to human sensory results since the E-nose mimics the human olfactory system [25,26,28]. In the chemometric approaches, although furan derivatives are structurally a subset of heterocyclic compounds, they were categorized separately due to their unique sweet and caramellic flavor traits characteristic of *O. fragrans*. This classification is clearly reflected in the PCA-biplot (Figure 3), where furan derivatives and other heterocycles exhibit distinct loading trajectories along the PC2 axis, demonstrating their differentiated contribution to sample clustering. Previous studies have indicated that the type of olfactory receptor binding to volatile compounds depends on concentration. The variation can result in different odor activation and description compared to those at lower concentrations [7,12]. The functions of these olfactory receptors play a key role in odor perception, as well as various physiological roles, including appetite regulation, energy metabolism, muscle regeneration, and inflammation [13]. Additionally, previous studies reported that the physiological effects of volatiles inhalation in vivo test (rats) were identified between low and high concentrations. The results revealed that positive effects, which include reduced visceral fat mass and upregulated cholesterol levels, were identified at the lower concentration; however, the negative effects, which include accelerated visceral fat accumulation, were represented at the higher concentration [5,8]. In this study, the four clusters were identified using PCA and HCA based on the E-nose data, and 1%, 3%, and 5% *O. fragrans* extracts were represented as cluster IV. Particularly, 3% and 5% *O. fragrans* extracts were represented as the sub-cluster B. Considering the results of E-nose and chemometric approaches and previous studies, 3% and 5% concentrations of *O. fragrans* extracts were classified as a different cluster compared with DW. Additionally, these extracts showed a relatively low concentration among all concentrations of *O. fragrans*. Therefore, the optimal inhalation concentrations can be regarded as 3% and 5% *O. fragrans* extracts to minimize the potential side effects that could occur at higher concentrations. Additionally, individual volatile compounds generally bind to olfactory receptors in proportion to their concentration, and an increased concentration of volatile compounds binds to a higher number of olfactory receptors. Generally, high concentrations of volatile compounds can cause off-odors, unlike low concentrations. Furthermore, a previous study reported that a relatively low concentration of volatiles induced positive biological functions (reduced visceral fat mass and LDL-cholesterol level); however, a relatively high concentration of volatiles induced side effects of biological functions (increased visceral fat mass and blood lipid-related levels) [5].

EEG technique is used to observe human brain activity directly, and EEG signals are generated from the interaction between neuron and neuron [22]. In detail, EEG signals are mainly classified as the five EEG frequency bands, including delta, theta, alpha, beta, and gamma waves [15,22]. Furthermore, alpha and beta waves can be separated according to the frequency range. Alpha waves can be classified as slow and fast alpha waves, and beta waves can be classified as low-, mid-, and high-beta waves [29]. These EEG waves are highly associated with psychological status and/or mental condition [21,29]. Changes in EEG waves, 5% inhalation of *O. fragrans* showed significantly increased RA and decreased RB, RG, RLB, and RHB waves in males (*p* < 0.05). Similar to male results, 5% inhalation of *O. fragrans* showed significantly increased RA and RSA, and significantly decreased RB, RLB, RMB, and RHB waves in females (*p* < 0.05). Previous studies reported that RA and RSA waves are highly associated with relaxation, calm, and rest states. However, RLB, and RMB waves are highly associated with an active state, which includes awareness and attention [22,29,30,31]. Additionally, RHB and RG waves are highly associated with a mental strain state, which includes tension, stress, anxiety, and a high degree of cognitive action [22,29]. Previous studies reported that alpha and gamma frequency bands played a key role in distinguishing the chemosensory stimuli [32,33,34]. In this study, discrimination among before, 3%, and 5% *O. fragrans* odor stimuli elicited EEG activities predominantly in the RA and RG frequency ranges. Additionally, the neurophysiological effects on human EEG were identified via 5% inhalation of *O. fragrans*, and olfactory stimulation activated relaxation-related EEG waves (RA) and reduced anxiety and tension-related EEG waves (RG), regardless of gender differences. Accordingly, *O. fragrans* inhalation may induce mental and physical stability. Compared with gender differences, inhalation concentrations between 3% and 5% have played a significant role in variations in EEG activities.

Brain responses can be reflected through an EEG signal, which effectively interprets a variety of chemosensory and psychological states [14]. Despite its advantages, EEG data can only provide limited data, such as topography data and limited brain region data (pre-frontal, frontal, temporal, parietal, and occipital lobes). Above these reasons, the utilization of sLORETA is necessary for the measurement of localized EEG signal sources and intracerebral activity [24]. sLORETA is used to investigation of Brodmann area (localized sources) and is based on the statistical non-nonparametric maps (SnPM) method through 5000 random trials (permutation). SnPM is one of the statistical methods of reasoning and provides statistical inferences based on non-parametric randomization/permutation tests at the level of voxel in the functional brain imaging experiments [22,24,35,36]. The results of this study indicated that sLORETA was conducted between voxels (before vs. 3% *O. fragrans* inhalation) and voxels (before vs. 5% *O. fragrans*), and a total of two EEG frequency bands (RHB and RG) were significantly changed after the inhalation of 5% *O. fragrans*. Following 5% *O. fragrans* inhalation, the insula in the sub-lobar and the precentral gyrus in the frontal lobe were significantly deactivated in RHB and RG waves of male participants. Among these intracerebral regions and EEG waves, the insula (Brodmann areas 13) in the sub-lobar and superior temporal gyrus (Brodmann area 22) in the temporal lobe were significantly higher changes compared with the precentral gyrus (Brodmann areas 44) in the frontal lobe and sub-gyral (Brodmann area 21) in the temporal lobe at RHB and RG waves after inhalation of 5% *O. fragrans* (*p* < 0.01 vs. *p* < 0.05). Previous studies reported that between RHB and RG waves, these EEG waves are commonly responsible for the mental statin status, including anxiety, stress, and a high degree of cognitive status [22,24,29]. Among significantly variations in intracerebral regions (Brodmann areas), inferior frontal gyrus (Brodmann area 44) is related to language and speech processing in the Broca’s area [24,37,38,39]. Middle temporal gyrus (Brodmann areas 21 and 22) is related to semantic memory processing, language, visual perception, and multi-modal sensory integration [24,40]. The insula (Brodmann area 13) is related to a higher cognitive- and emotional functions, language processing [41,42]. Particularly, these Brodmann areas were only changed in male participants. Accordingly, the localized EEG-signal sources were significantly decreased in RHB and RG waves of male participants after inhalation of 5% *O. fragrans* extracts (*p* < 0.05). In this study, 3% and 5% concentrations of *O. fragrans* were not distinguished through the E-nose system; however, these different concentrations of *O. fragrans* were distinguished through human EEG measurement. Accumulating evidence indicates that biological sex significantly modulates olfactory sensitivity and subsequent emotional processing due to dimorphic receptor expressions and endocrine profiles [24,43,44,45]. Based on this, we hypothesized that males and females would exhibit distinct neural oscillation patterns in specific EEG frequency bands and localized brain regions upon exposure to *O. fragrans* volatiles. Despite these meaningful insights, some methodological limitations must be addressed. Although a humidifier was utilized, the actual airborne concentration and real-time diffusion rate of the volatiles were not dynamically quantified, and environmental parameters like room volume, ventilation, and ambient conditions were not systematically monitored. Consequently, the precise inhalation dosage remains unquantified, potentially introducing confounding variables into the neurophysiological outcomes. Future investigations should therefore utilize advanced olfactometers within strictly regulated environmental chambers for precise exposure metrics.

## 4. Materials and Methods

### 4.1. Ethical Approval

This study design and all procedures were approved by the Korea National Institute for Bioethics Policy IRB (approval No. P01-202101-13-003). Furthermore, all participants have consented to participate in this experiment.

### 4.2. Sample

The flowers of *O. fragrans* were collected at the full-flowering stage in Gyeongnam province in Republic of Korea. During the extraction processes, 1.5 g of *O. fragrans* and 15 mL of 95% ethanol solvent were mixed for 24 h and the mixtures were stored at 4 °C [2]. In addition, the ethanol extract of *O. fragrans* was diluted with DW according to five concentrations (1%, 3%, 5%, 7%, and 9%) for E-nose analysis.

### 4.3. Electronic Nose Analysis

The volatile compound profiles in the *O. fragrans* extract were analyzed using an E-nose (HERACLES Neo, Alpha Mos Co., Toulouse, France). A 6 mL of *O. fragrans* extract was placed in a headspace vial (22.5 × 75 mm, PTFE; polytetrafluoroethylene) with a silicone septum and aluminum cap. The samples were the six kinds of extracts, including (DW) 1%, 3%, 5%, 7%, and 9% *O. fragrans* extracts. The volatiles in the headspace of the vial were collected for 10 min at 50°C (stirring process), facilitated by an incubator within the E-nose system. Following the collection of volatile compounds in the headspace, an additional 5000 μL of volatile compounds were collected using an automatic sampler connected to the E-nose system. The system utilized a flame ionization detector (FID; Alpha MOS Co., Toulouse, France) and an MXT-5 column (2 m × 0.18 mm, 5% diphenyl/95% dimethyl polysiloxane, Alpha MOS Co., Toulouse, France). The FID was set at a temperature of 260°C, with a hydrogen flow rate of 1 mL/min. The sample acquisition time was set at 277 s, with trap absorption and desorption temperatures set at 40 °C and 250 °C, respectively. The initial oven temperature was held at 40 °C for 5 s, then increased to 270 °C at a rate of 4 °C/s, and maintained for 30 s. For the qualitative analysis of the E-nose, a headspace injection solution (C_6_–C_16_) of 20 uL was introduced into the E-nose. Each volatile compound was identified using Kovat’s index (the retention index in terms of carbon number) through the library-based AcroChembase (Alpha Mos Co.), and the volatile compounds corresponding to each peak were subsequently analyzed [25].

### 4.4. Participant Characteristics and Experimental Designs

In this study, a total of 20 participants, including 10 males and 10 females, were involved. Inclusion criteria were defined as the subjects, which were randomized, with the chosen group consisting of 20 individuals generally in their twenties. Exclusion criteria were defined as the subjects with low olfactory performance, olfactory dysfunction, and smoking. Furthermore, all subjects exhibited normal blood pressure levels before inhalation. Before the experiment, subjects were instructed to abstain from caffeine and alcohol intake for a duration exceeding 16 h. Additionally, detailed information about participants is shown in Table 3. Following a stabilization period of 15 min for each participant, measurements of EEG were conducted before and during the inhalation process. For 5 min inhalation of 3% and 5% *O. fragrans* extracts, a humidifier (Aroma diffuser humidifier, Cactus Co., Shenzhen, China) was positioned approximately 15 cm from the subject’s nose [22]. The 3% and 5% concentrations were strategically selected based on initial E-nose clustering, representing a lower-concentration group distinct from distilled water. This selection aimed to prevent receptor saturation and potential adverse effects during human exposure. All participants received detailed instructions, and they were provided with informed consent before commencing the experiments and received monetary compensation based on their contribution. All human experimental procedures were approved by the Korea National Institute for Bioethics Policy IRB (approval No. P01-202101-13-003) and were performed in accordance with the code of ethics human experimentation of the World Medical Association (Declaration of Helsinki).

### 4.5. Human EEG Measurement

**Figure 9:**
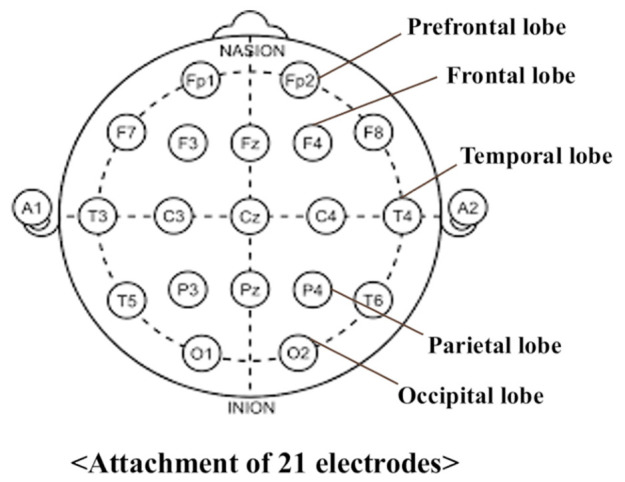
The attachment sites of 21 electrodes.

**Figure 10:**
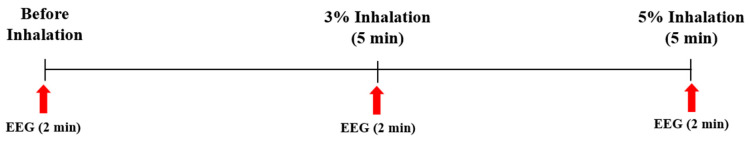
Experimental design for EEG analysis.

An EEG measuring device (S-24 EEG; Biobrain Co., Daejeon, Republic of Korea) was utilized, and an electrode was affixed to the scalp using a disk electrode paste (ElfixZ-401CE; Biobrain Co.). The electrodes of the EEG cap were positioned at 21 locations (Figure 9), including the prefrontal, frontal, temporal, parietal, and occipital lobes. The subject’s eyes were closed, and an EEG was conducted for 2 min in a relaxed state (control value), followed by another 2 min after 3% and 5% concentrations of inhalation (experimental values) (Table 4 and Figure 10). In detail, after the olfactory stimulation of the 3% extract and subsequent EEG measurement, a 30 min washout period was strictly implemented in a well-ventilated, odorless room to eliminate any potential carryover or adaptation effects before the 5% extract inhalation. Data was collected and scrutinized using the EEG program (Bios single program, Biobrain Co.). Individual data were represented as the ratio of the experimental value to the control value. The sampling rate was maintained at 256 Hz with an online impedance control criterion strictly kept below 5 kΩ across all active channels. To eliminate environmental and physiological noise, a digital band-pass filter of 0.5–50 Hz and a 60 Hz notch filter were applied. Gross muscle artifacts, body movements, and severe eye-blink segments were primarily minimized during the recording session by instructing participants to remain still with their eyes closed. During the offline preprocessing phase using the BioScan software (version 11) platform, the continuous EEG stream was visually inspected. Any abnormal segments, sudden voltage spikes exceeding ±200 μV, or technical artifacts were strictly rejected manually to ensure that only artifact-free, stationary EEG data were advanced to spectral analysis. For each experimental condition, a stabilized, artifact-free continuous window of 2 min was extracted and utilized as the effective analysis duration. The power spectrum was segmented into four standard frequency bands defined by the analysis system: Theta (4–8 Hz), Alpha (8–13 Hz), Beta (13–30 Hz), and Gamma (30–50 Hz). In detail, alpha wave was separated into slow alpha (8–11 Hz) and fast alpha (11–13 Hz) waves, and beta wave was separated into low beta (13–15 Hz), mid beta (15–20 Hz), and high beta (20–30 Hz) waves, respectively. The relative power for each specific band was calculated via the system’s Batch Mapping script by dividing the absolute power of the respective band by the total power integrated across the entire spectrum (0.5–50 Hz). This normalized index was used to construct the final topographic brain maps. Based on the EEG signal data, the brain mapping program (EEG brain mapping; Biobrain Co.) was employed to ascertain the mean EEG value of the participants [22].

### 4.6. sLORETA

**Figure 11:**
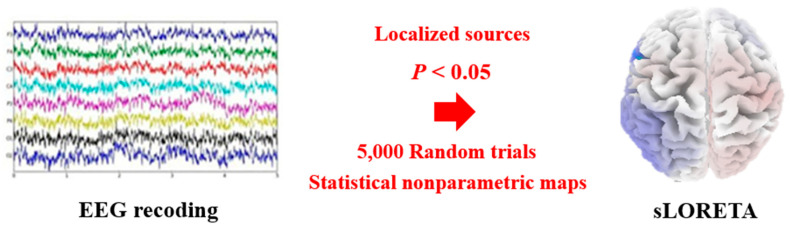
The processing of intracerebral signals through standardized low-resolution brain electromagnetic tomography.

sLORETA approach was performed according to sex differences, including RT, RA, RSA, RFA, RB, RLB, RMB, RHB, and RG band frequency. Brain-localized sources (intracerebral signals) were investigated and identified through the inverse problem based on EEG data. During the sLORETA procedure, each text file containing EEG data collected after inhalation of 3% *O. fragrans* and after inhalation of 5% *O. fragrans* was converted into a ‘slor’ file for each participant, followed by the execution of a corresponding sample test (paired group test, A–A2 = B–B2). Additionally, A2 and B2 were designated as eye-open states, while a corresponding sample test was selected to ascertain the differences in Brodmann areas (brain activation) after 3% and 5% *O. fragrans* inhalation within the same participant, respectively. The statistical differences between voxels across nine frequency bands (RT – RG) were identified by projecting the statistical non-parametric mapping (SnPM) method through 5000 random trials (permutation; simulation) (Figure 11). A paired-sample *t*-test (paired group contrast) was executed within the sLORETA software (version 20240713) environment to statistically compare the voxel-by-voxel current density distributions between the two experimental conditions (pre-inhalation resting state vs. 3% inhalation and pre-inhalation resting state vs. 5% inhalation). The input data consisted of cross-spectral matrices derived from artifact-free, preprocessed EEG segments via Fast Fourier Transform (FFT). To robustly control for Type I errors without rigid parametric assumptions, Statistical Non-Parametric Mapping (SnPM) was implemented. The critical threshold values for the *t*-statistic were determined using a randomization/permutation test with 5000 permutations at a significance level of *p* < 0.05. This non-parametric randomization approach inherently corrects for multiple comparisons across all 6239 voxels and frequency bands simultaneously (Family-Wise Error [FWE] correction). Source localization was computed using a realistic head model based on the Montreal Neurological Institute (MNI152) template. The solution space was restricted to the cortical gray matter and hippocampus, consisting of 6239 voxels at a spatial resolution of 5 × 5 × 5 mm. The generated MNI coordinates were automatically converted into Talairach coordinates within the LORETA-KEY software (version 20240713) suite to provide precise identification of anatomical structures and corresponding Brodmann Areas (BAs) [24].

### 4.7. Data Processing

The results of the present study were represented as mean ± standard deviations, and significant differences among EEG signal variations were identified through the Duncan test using SAS version 9.0 (SAS Institute Inc., Cary, NC, USA) package (*p* < 0.05). The visual representation of the E-nose and EEG data was obtained using chemometrics, which include principal component analysis (PCA), hierarchical cluster analysis (HCA). These chemometric approaches were employed to discern patterns and dendrograms among samples and variables. For the execution of chemometric approaches, PCA, and HCA were conducted using XLSTAT software (version 9.2, Addinsoft, New York, NY, USA) [26].

## 5. Conclusions

This study investigated the volatile compound profiles of both DW and *O. fragrans* extracts using an E-nose system. In E-nose results, a total of 48 volatiles were identified, and hydrocarbons, alcohols, acids, and esters were the dominant compounds. The contents of alcohols in *O. fragrans* extracts increased in a concentration-dependent manner. In the results of chemometric approaches, PCA and HCA showed distinct patterns among different concentrations of *O. fragrans* extracts by different distributions (PCA) and clusters (HCA). Interestingly, the chemometric approaches of E-nose data revealed that concentrations of 3% and 5% *O. fragrans* were identified as optimal conditions. In the EEG evaluation, the effects of *O. fragrans* inhalation on human brain activity were examined. Significant changes in EEG waves were observed, including an increase in sedation-related waves (RA and RSA) and a decrease in tension-related waves (RB, RG, RLB, and RHB) resulting from inhalation of *O. fragrans* in male and female participants. Especially, these inhalation effects were mainly represented by the 5% inhalation of *O. fragrans*. Furthermore, these effects did not show any significant variations according to the sex differences. In sLORETA results, inhalation of *O. fragrans* induced the significant changes in RHB and RG waves identified in male participants (*p* < 0.05), and a total of four Brodmann areas (13, 21, 22, and 44) were significantly decreased after 5% O. *fragrans* inhalation. Additionally, quantifiable data on human brain response and volatile compound profiles were also identified using biomimetic-based olfactory and EEG techniques combined with chemometrics and permutation. The 5% concentration of *O. fragrans* extract elicited a more pronounced modulation of neural activity compared to the 3% concentration. Furthermore, unlike the E-nose measurements, EEG analysis successfully discriminated between the 3% and 5% concentrations in both male and female participants. Ultimately, this study successfully established a comparative framework bridging machine perception (via E-nose profiling) and human olfactory cognition (via quantified EEG analysis) combined with chemometrics and permutation. Although the flash GC-based E-nose provided a robust volatile fingerprint for this purpose, the lack of traditional GC-MS characterization for absolute quantification remains a limitation, which will be integrated in future studies to further elucidate specific compound-neural relationships. Additionally, this study has some limitations, including a small sample size and the absence of controls, and this study demonstrated short-term EEG alterations rather than definitive sedative or therapeutic efficacy. Further study will require larger controlled cohorts, long-term clinical evaluation, and the integration of subjective behavioral metrics.

## Figures and Tables

**Table 1:** Volatile compound profiles in different concentrations of *Osmanthus fragrans* extracts using E-nose (Peak area × 10^3^).

Volatile Compounds	RT ^(1)^ (RI ^(2)^)	Sensory Description	Distilled Water	1%	3%	5%	7%	9%
**Acids and Esters (9)**								
Methyl acetate	19.06(526)	Blankcurrant, Fruity, Sweet	ND ^(3)^	ND	1.11 ± 0.15	ND	ND	ND
Butanoic acid	43.13(800)	Butter, Cheese, Sweaty	ND	0.41 ± 0.06	ND	ND	1.65 ± 0.06	ND
Methyl pentanoate	45.97(827)	Apple, Fruity, Green, Nutty	ND	ND	ND	1.26 ± 0.13	ND	ND
Butyl 2-propenoate	53.87(903)	-	ND	ND	ND	0.28 ± 0.03	ND	ND
Pentanoic acid	54.01(905)	Beefy, Cheese, Sweaty, Sour	ND	ND	ND	ND	ND	0.43 ± 0.03
Ethyl pentanoate	54.14(906)	Apple, Fruity, Orange, Sweet	ND	ND	0.41 ± 0.26	ND	ND	ND
2-Butenoic acid, pentyl ester	71.60(1142)	-	265.00 ± 122.98	ND	ND	ND	ND	ND
Molinate	91.07(1524)	Aromatic	ND	ND	ND	ND	12.48 ± 0.40	ND
Isopropyl cinnamate	91.19(1527)	Balsamic	ND	ND	ND	9.74 ± 0.50	ND	ND
**Alcohols (12)**								
Ethanol	14.96(435)	-	ND	84.35 ± 3.68	301.63 ± 23.76	392.34 ± 20.51	560.61 ± 58.07	703.14 ± 43.26
Methanethiol	16.34(466)	Cabbage, Cheese, Egg, Garlic	99.80 ± 35.33	ND	ND	ND	ND	ND
2-Propanol	17.77(498)	Acetone, Musty, Woody	ND	ND	ND	ND	2.35 ± 0.38	ND
1-Propanol	20.21(552)	Fruity, Musty	ND	ND	0.16 ± 0.07	ND	ND	ND
2-Mercaptoethanol	20.31(554)	-	ND	ND	1.88 ± 0.13	0.10 ± 0.09	0.19 ± 0.06	0.43 ± 0.33
2-Butanol	22.36(600)	Sweet, Wine	69.68 ± 65.53	ND	ND	ND	ND	ND
Butanol	27.13(654)	Banana, Cheese, Fruity	ND	ND	ND	ND	ND	0.08 ± 0.03
Butanethiol	34.23(725)	Coffee, Garlic, Onion	ND	ND	ND	ND	ND	0.15 ± 0.03
Prenyl mercaptan	45.11(818)	Onion, Roast, Smoky	ND	ND	ND	ND	ND	2.10 ± 0.03
Phenol	62.68(1008)	Aromatic, Sweet	174.81 ± 68.89	ND	ND	ND	ND	ND
Dimethylphenol	70.62(1125)	Coffee, Rooty, Sweet	ND	ND	ND	ND	ND	2.69 ± 0.17
Maltol	71.61(1142)	Baked, Caramelized	ND	ND	ND	ND	2.47 ± 0.35	ND
**Aldehydes (4)**								
2-Methylpropanal	19.10(527)	Baked potato, Green, Fruity	ND	ND	ND	ND	1.79 ± 0.20	ND
Butanal	21.42(579)	Chocolate, Cocoa, Green, Musty	ND	ND	ND	ND	ND	4.30 ± 0.49
2-Butenal	27.09(653)	Floral, Green	ND	ND	ND	ND	0.19 ± 0.02	ND
2-Methylbutanal	28.19(665)	Almond, Apple, Cocoa, Coffee	ND	0.59 ± 0.05	1.91 ± 0.12	2.45 ± 0.12	3.48 ± 0.11	ND
**Hydrocarbons (17)**								
Trimethylamine	15.04(436)	Fishy, Fruity, Oily	110.82 ± 26.97	ND	ND	ND	ND	ND
Dichloromethane	17.70(496)	Sweat	ND	0.41 ± 0.10	ND	1.71 ± 0.33	ND	ND
1,2-Dichloroethene	19.02(526)	Mild, Sweet	ND	0.36 ± 0.03	ND	ND	ND	ND
Hexane	22.41(601)	-	ND	ND	ND	2.37 ± 0.16	ND	ND
Methylcyclopentane	23.84(617)	-	ND	ND	ND	0.74 ± 0.03	0.99 ± 0.03	1.27 ± 0.12
Chloroform	23.87(617)	-	ND	ND	0.59 ± 0.13	ND	0.97 ± 0.06	ND
Heptane	30.19(688)	Fruity, Sweet	ND	ND	0.37 ± 0.08	ND	ND	ND
2,2,4-Trimethylpentane	30.21(688)	-	ND	ND	ND	0.48 ± 0.04	0.59 ± 0.18	ND
Octane	43.36(802)	Fruity, Sweet	ND	ND	1.24 ± 0.14	ND	ND	ND
Decane	62.16(1001)	Fruity, Sweet	ND	ND	ND	ND	16.37 ± 0.74	ND
5-Methyl-4-nonene	63.38(1018)	-	ND	2.63 ± 0.12	ND	ND	ND	ND
*α*-Phellandrene	63.39(1019)	-	ND	ND	ND	12.29 ± 0.27	ND	ND
Dipentene/Terpene/limonene	64.03(1028)	Citrus, Green, Pine	ND	ND	9.88 ± 0.59	ND	ND	ND
*p*-Menthatriene	69.53(1107)	Turpentine, Woody	ND	ND	ND	1.74 ± 0.09	ND	ND
Undecane	69.60(1109)	-	ND	0.57 ± 0.15	ND	ND	ND	ND
Limonene oxide	71.33(1137)	Citrus, Fresh, Minty, Floral	ND	ND	1.50 ± 0.05	ND	ND	ND
2,4-Dinitrotoluene	91.27(1529)	-	2311.36 ± 853.86	3.21 ± 0.21	ND	ND	ND	ND
**Ketones (4)**								
Propan-2-one	17.71(496)	Apple, Fruity, Pear, Sweet	ND	ND	1.40 ± 0.27	ND	ND	3.17 ± 0.45
1-Hydroxy-2-propanone	27.07(653)	Caramelized, Sweet	ND	ND	0.05 ± 0.04	ND	ND	ND
Acetoin	34.12(724)	Butter, Coffee, Creamy, Milky	ND	ND	ND	ND	0.10 ± 0.05	ND
2-Acetyl-1-pyrroline	55.91(927)	Cracker, Meat, Nutty, Roast	ND	ND	ND	ND	0.14 ± 0.02	ND
**Heterocyclic compound (1)**								
Vinylpyrazine	56.75(937)		ND	ND	ND	ND	ND	0.09 ± 0.05
**Furan (1)**								
2-Butylfuran	54.01(905)	Fruity, Mild, Spicy, Sweet	ND	ND	ND	ND	0.36 ± 0.02	ND

^(1)^ RT: retention time. ^(2)^ RI: retention index. ^(3)^ ND: not detected.

**Table 2:** Changes in Brodmann area after inhalation of 5% *O. fragrans*.

Sex	EEG Frequency Band	MNI Coordinates			Voxel Value	Lobe	Structure	Brodmann Area
		X	Y	Z	(maxLOR)			
Male	Relative high beta	45	−5	0	−8.19	Sub-lobar	Insula	13
		50	−5	0	−7.18	Temporal Lobe	Middle Temporal Gyrus	22
		50	−5	0	−5.91	Frontal Lobe	Inferior frontal gyrus	44
		45	−5	−10	−5.56	Temporal Lobe	Middle Temporal Gyrus	21
	Relative gamma	45	−5	0	−8.19	Sub-lobar	Insula	13
		50	−5	0	−7.18	Temporal Lobe	Middle Temporal Gyrus	22
		50	−5	0	−5.91	Frontal Lobe	Inferior frontal gyrus	44
		45	−5	−10	−5.56	Temporal Lobe	Middle Temporal Gyrus	21
Female	Relative high beta	-	-	-	-	-	-	-
		-	-	-	-	-	-	-
		-	-	-	-	-	-	-
		-	-	-	-	-	-	-
	Relative gamma	-	-	-	-	-	-	-
		-	-	-	-	-	-	-
		-	-	-	-	-	-	-
		-	-	-	-	-	-	-

**Table 3:** Detailed information about participants.

Parameter/Category	Specification/Criteria	Value/Status
**Participant Demographics**		
Total Sample Size (*n*)	Healthy young adults	20
Sex Distribution	Male/Female	10 males/10 females
Age Range	Young adults in their general twenties	20–29 years
Mean Age	Mean ± SD	Males: 24.4 ± 1.4, Females: 23.5 ± 1.5
Physiological Status	Baseline blood pressure	Normal range
**Inclusion Criteria**		
Age and Target Group	Standardized healthy individuals in their twenties	Mandated
Olfactory Performance	Confirmed normal olfactory performance and acuity	Mandated
Baseline Health	Documented normal blood pressure levels prior to testing	Mandated
Pre-test Sleep	Mandatory normal sleep duration (>6 h) on the night prior to testing	Mandated
Informed Consent	Voluntarily provided written informed consent	Mandated
**Exclusion Criteria**		
Olfactory Dysfunction	Low olfactory performance, anosmia, or active nasal congestion	Excluded
Smoking History	Current smokers or history of recent smoking	Excluded
Medication History	Active intake of medications or history of neurological/psychiatric disorders altering Central nervous system or olfactory pathways	Excluded
Dietary Non-compliance	Failure to abstain from caffeine and alcohol for over 16 h prior to the experiment	Excluded

**Table 4:** Experimental design for the EEG analysis.

Group (General 20s)	State
Before	Before inhalation
(Males = 10, Females = 10)	
3% for 5 min	3% *O. fragrans* inhalation (5 min)
(Males = 10, Females = 10)	
5% for 5 min	5% *O. fragrans* inhalation (5 min)
(Males = 10, Females = 10)	

## Data Availability

The original contributions presented in this study are included in the article. Further inquiries can be directed to the corresponding author.

## Supplementary Materials

The following supporting information can be downloaded at: https://www.mdpi.com/article/10.3390/plants15132006/s1, Figure S1. Significant differences in EEG data on relative theta to high beta frequency bands in males and females (*p* < 0.05).
