# Supplementary material for: Neurophysiological Responses to Inhalation of Osmanthus fragrans Volatiles: A Combined Electronic Nose and Electroencephalogram (EEG) Study on Concentration-Dependent Effects
Source: Plants (Basel). 2026 Jun 29;15(13):2006. doi: 10.3390/plants15132006 (PMC13364178; doi:10.3390/plants15132006)
Supplement: Supplementary file 1 [file plants-15-02006-s001.zip › plants-4356342-supplementary.pptx]

## Slide 1
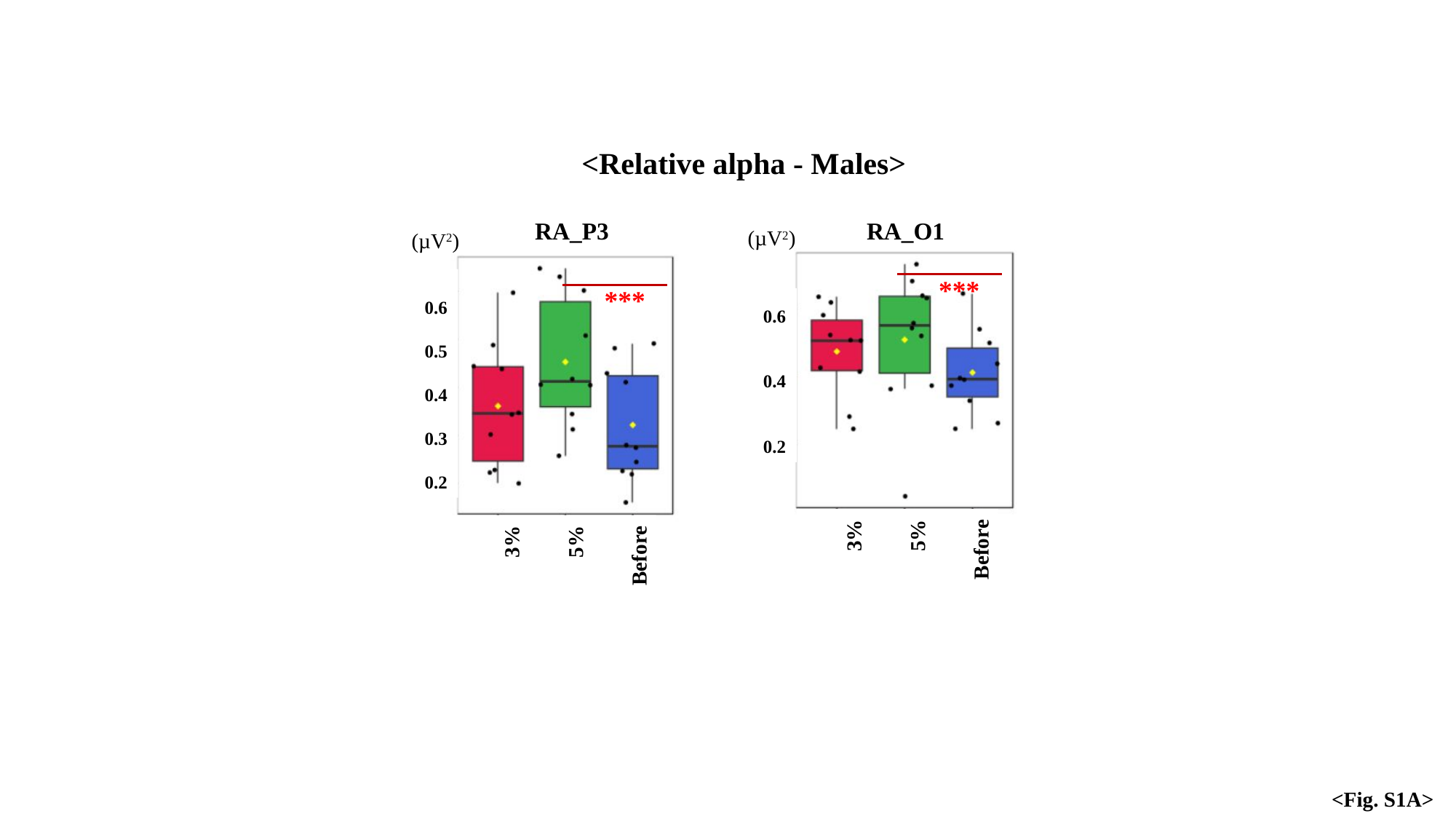

<Relative alpha - Males>
RA_P3
0.6
0.5
0.4
0.3
0.2
RA_O1
0.6
0.4
0.2
(µV2)
(µV2)
***
***
3%
5%
Before
3%
5%
Before
<Fig. S1A>

## Slide 2
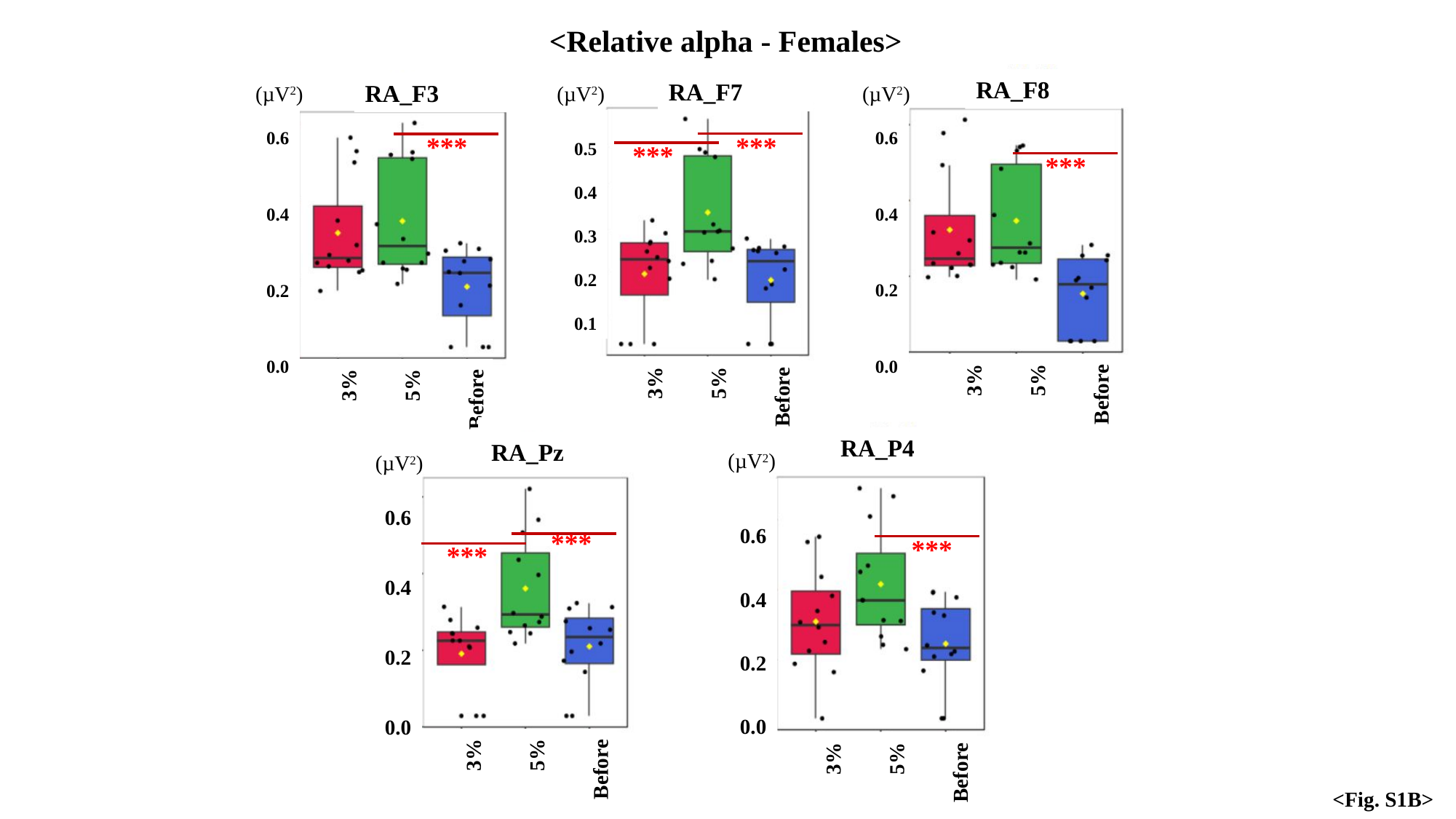

<Relative alpha - Females>
0.6
0.40.20.0
0.6
0.4
0.2
0.0
RA_F8
RA_F7
RA_F3
0.5
0.4
0.3
0.2
0.1
3%
5%
Before
(µV2)
(µV2)
(µV2)
***
***
***
***
3%
5%
Before
3%
5%
Before
RA_P4
RA_Pz
0.6
0.4
0.2
0.0
0.6
0.4
0.2
0.0
(µV2)
(µV2)
***
***
***
3%
5%
Before
3%
5%
Before
<Fig. S1B>

## Slide 3
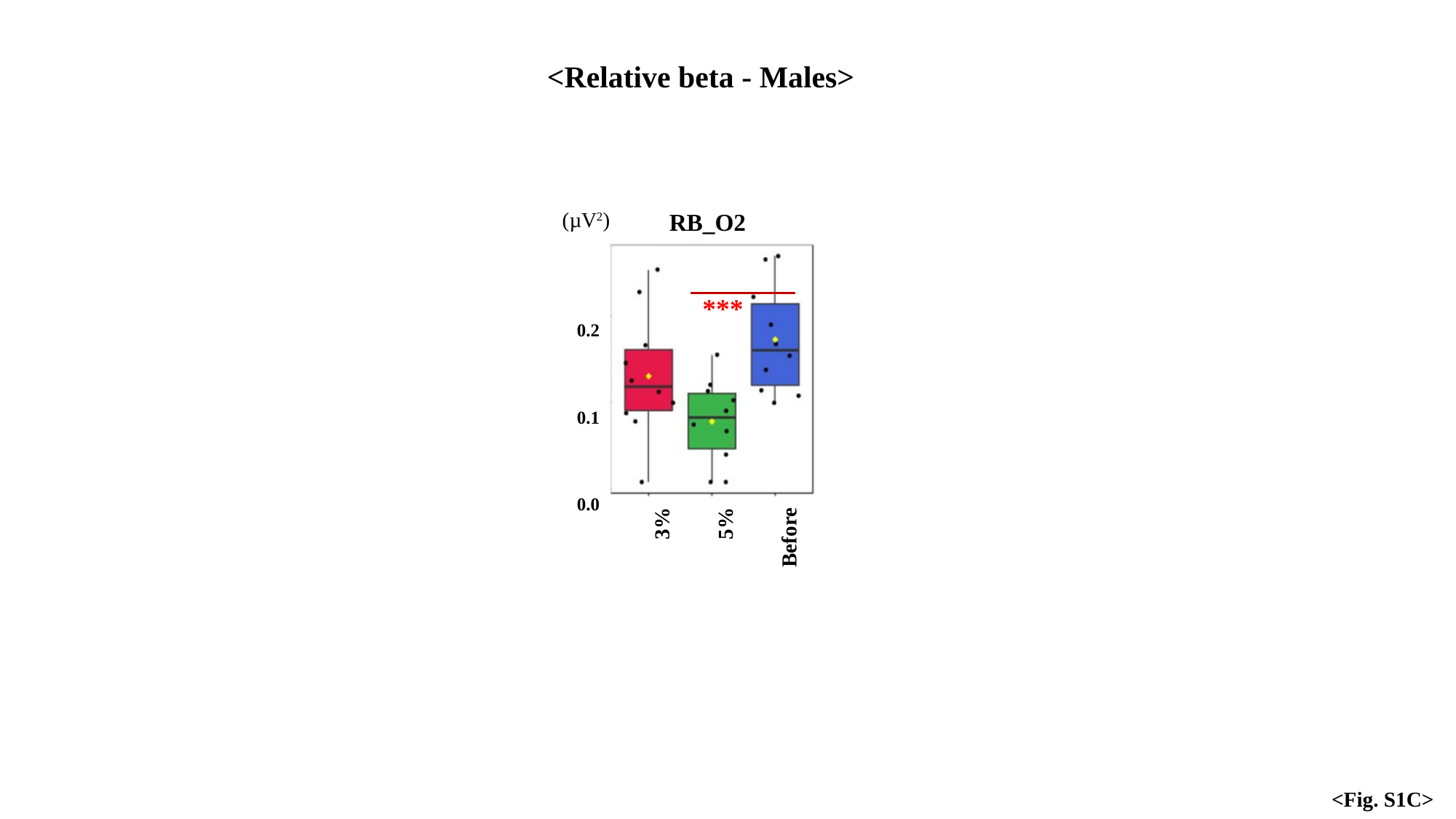

<Relative beta - Males>
(µV2)
RB_O2
0.2
0.1
0.0
***
3%
5%
Before
<Fig. S1C>

## Slide 4
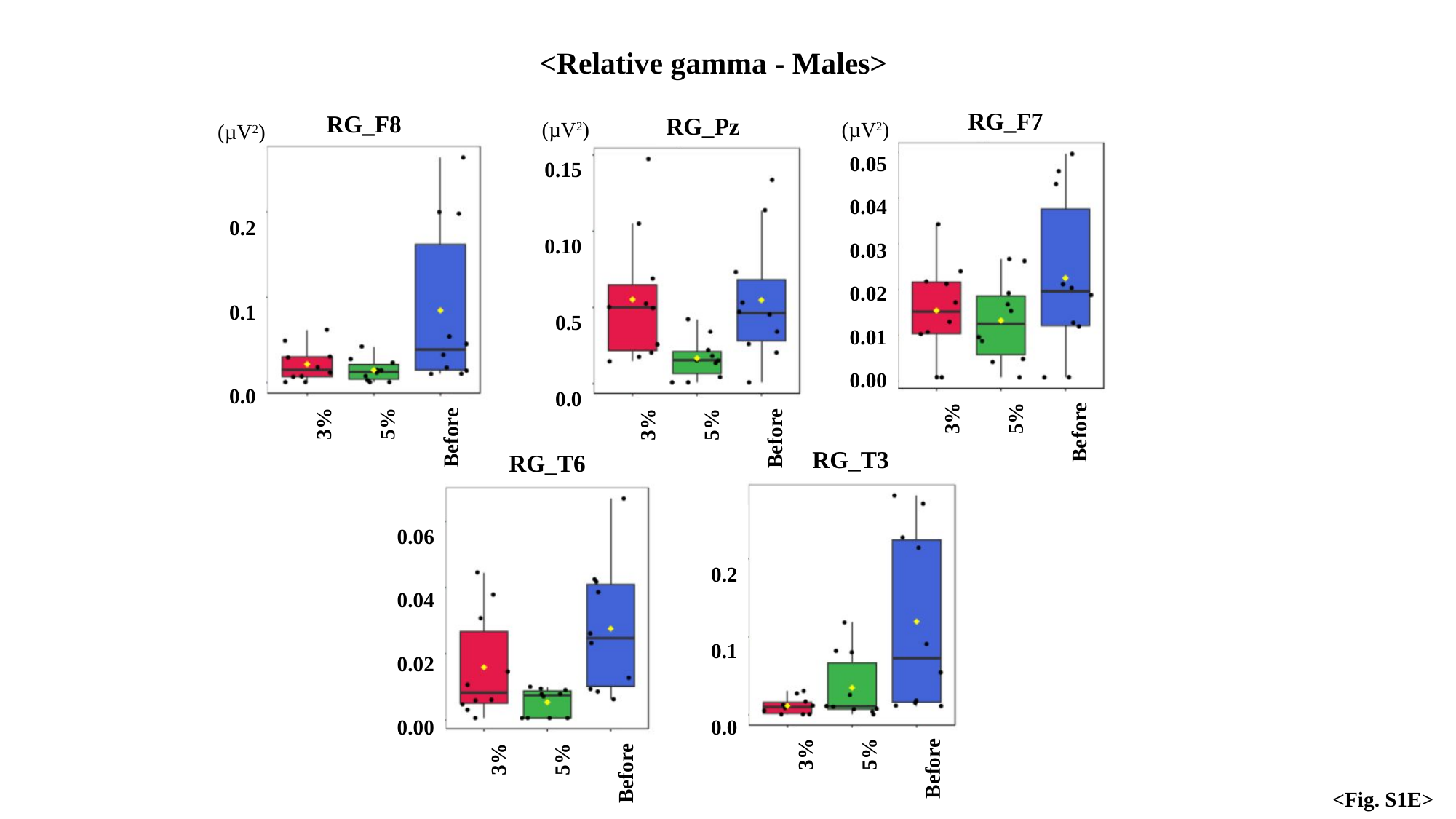

<Relative gamma - Males>
0.15
0.10
0.5
0.0
RG_F7
RG_F8
RG_Pz
0.05
0.04
0.03
0.02
0.01
0.00
0.2
0.1
0.0
3%
5%
Before
3%
5%
Before
3%
5%
Before
(µV2)
(µV2)
(µV2)
RG_T3
RG_T6
0.06
0.04
0.02
0.00
0.2
0.1
0.0
3%
5%
Before
3%
5%
Before
<Fig. S1E>

## Slide 5
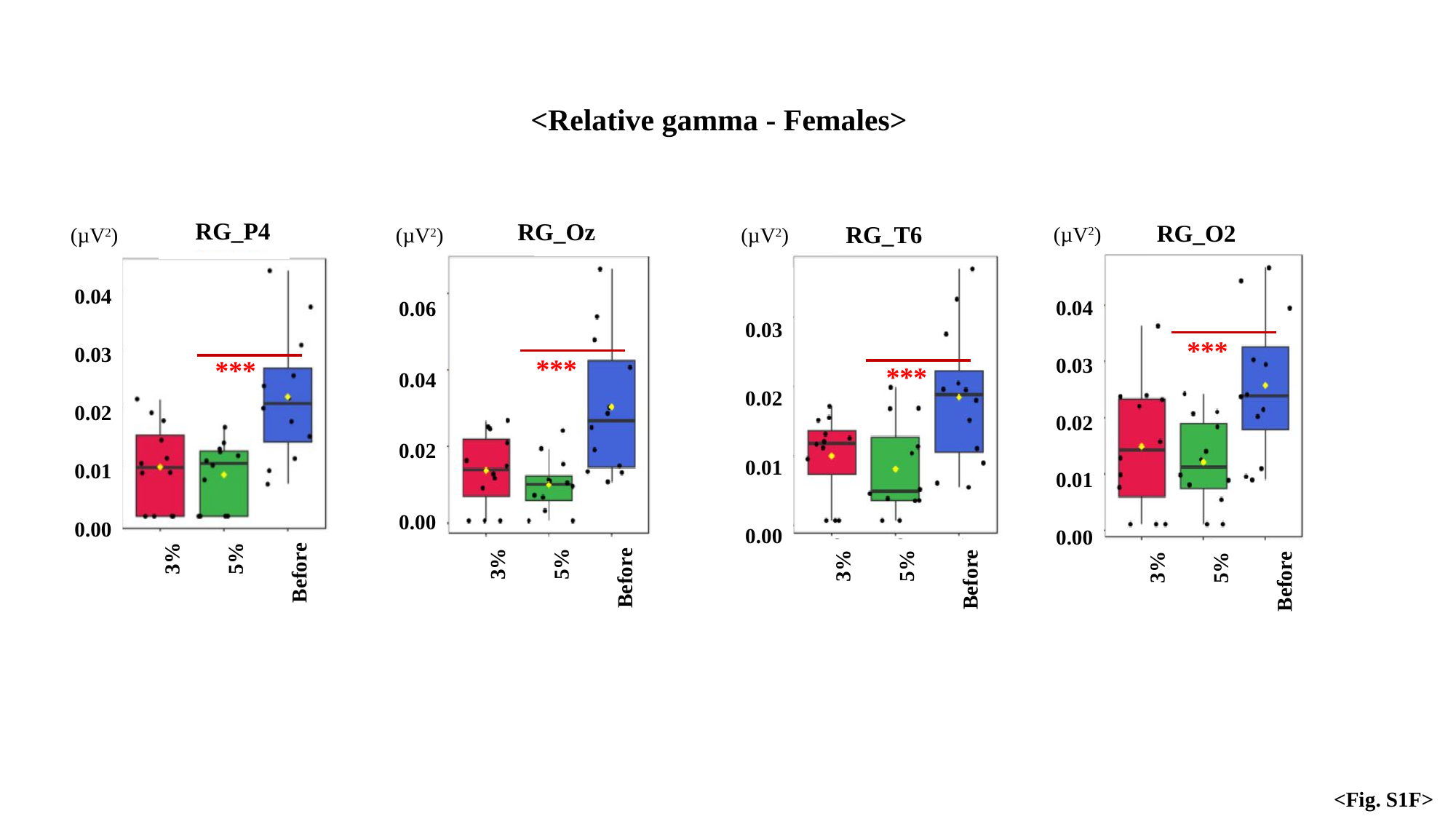

<Relative gamma - Females>
RG_P4
RG_Oz
RG_O2
RG_T6
0.06
0.04
0.02
0.00
0.04
0.03
0.02
0.01
0.00
0.04
0.03
0.02
0.01
0.00
0.03
0.02
0.01
0.00
3%
5%
Before
3%
5%
Before
3%
5%
Before
3%
5%
Before
(µV2)
(µV2)
(µV2)
(µV2)
***
***
***
***
<Fig. S1F>

## Slide 6
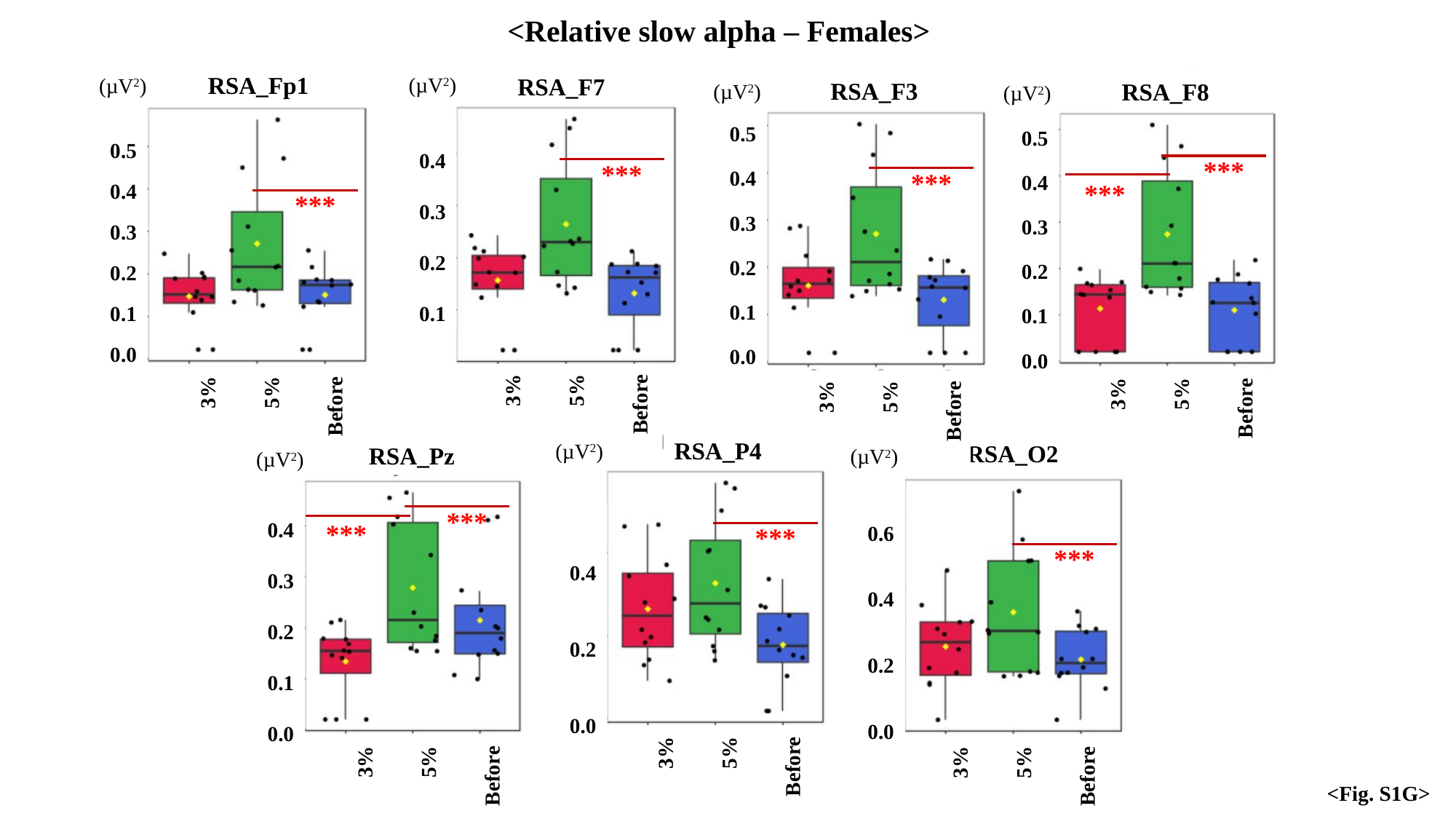

<Relative slow alpha – Females>
RSA_Fp1
RSA_F7
RSA_F3
RSA_F8
0.5
0.4
0.3
0.2
0.1
0.0
0.5
0.4
0.3
0.2
0.1
0.0
0.4
0.3
0.2
0.1
0.5
0.4
0.3
0.2
0.1
0.0
3%
5%
Before
3%
5%
Before
3%
5%
Before
3%
5%
Before
RSA_P4
RSA_O2
RSA_Pz
0.6
0.4
0.2
0.0
0.4
0.3
0.2
0.1
0.0
0.4
0.2
0.0
3%
5%
Before
3%
5%
Before
3%
5%
Before
<Fig. S1G>
(µV2)
(µV2)
(µV2)
(µV2)
***
***
***
***
***
(µV2)
(µV2)
(µV2)
***
***
***
***

## Slide 7
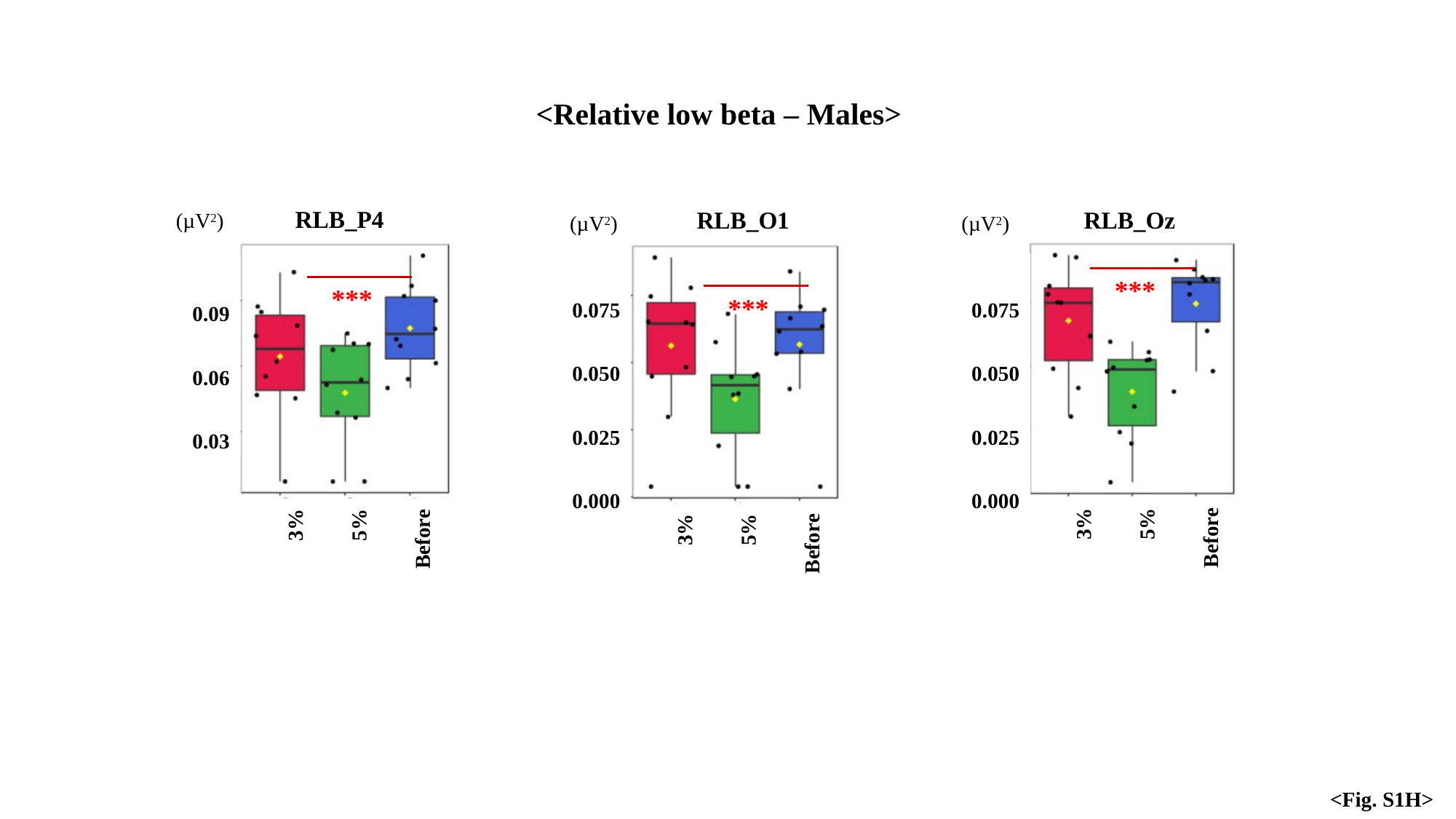

<Relative low beta – Males>
RLB_P4
RLB_O1
RLB_Oz
0.075
0.050
0.025
0.000
0.075
0.050
0.025
0.000
0.09
0.06
0.03
3%
5%
Before
3%
5%
Before
3%
5%
Before
(µV2)
(µV2)
(µV2)
***
***
***
<Fig. S1H>

## Slide 8
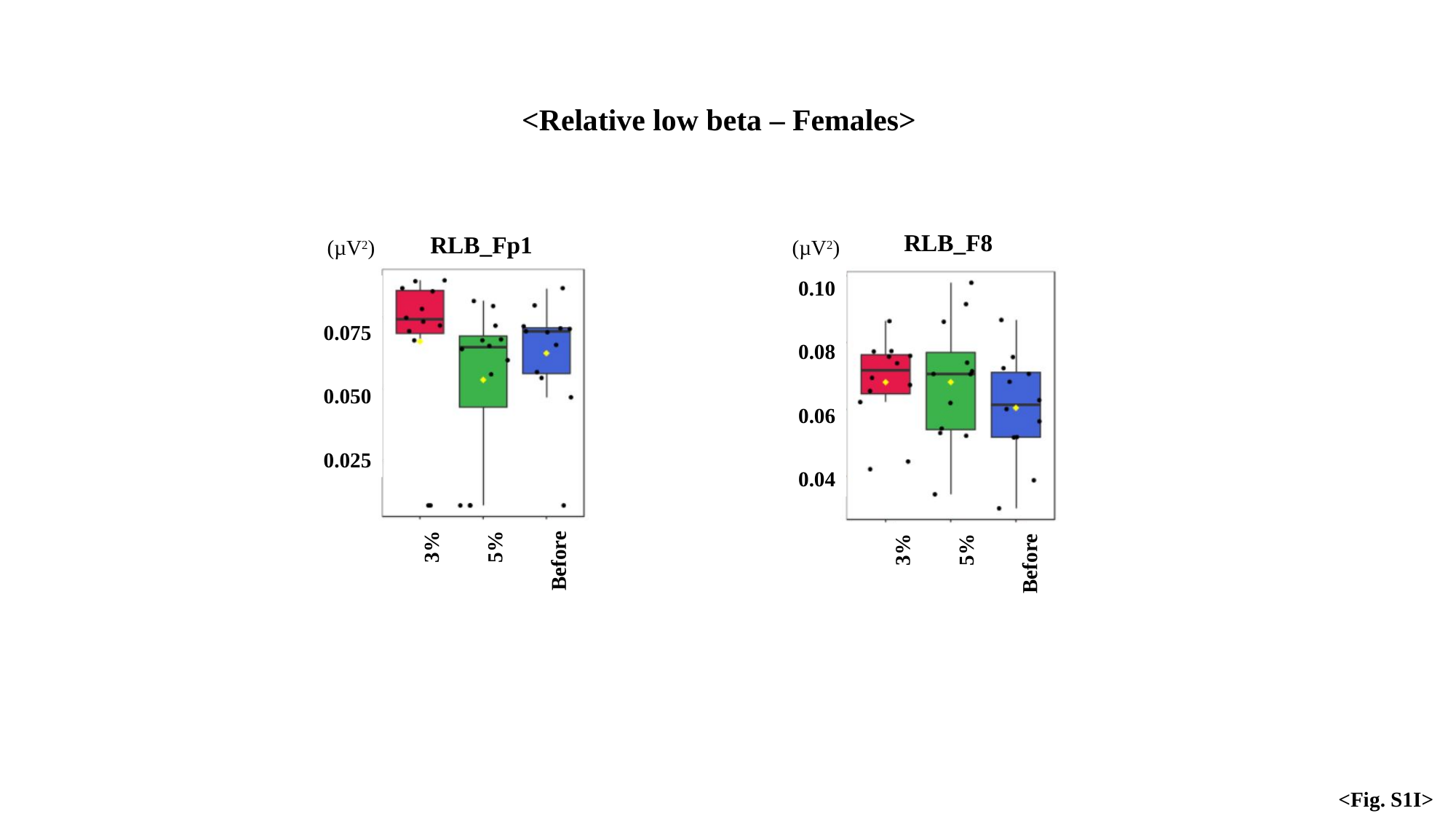

<Relative low beta – Females>
RLB_F8
RLB_Fp1
0.10
0.08
0.06
0.04
0.075
0.050
0.025
3%
5%
Before
3%
5%
Before
(µV2)
(µV2)
<Fig. S1I>

## Slide 9
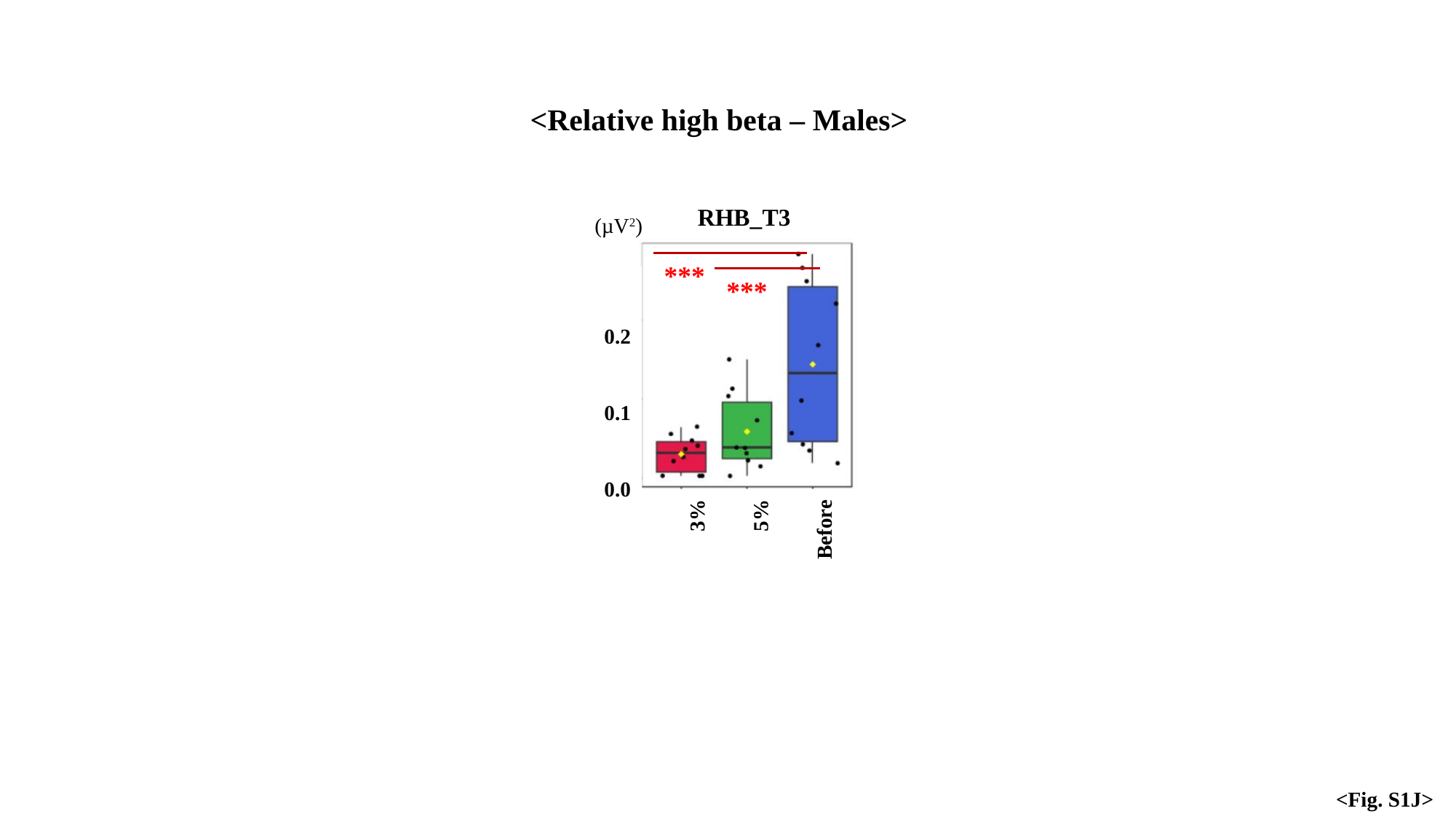

<Relative high beta – Males>
RHB_T3
0.2
0.1
0.0
3%
5%
Before
(µV2)
***
***
<Fig. S1J>

## Slide 10
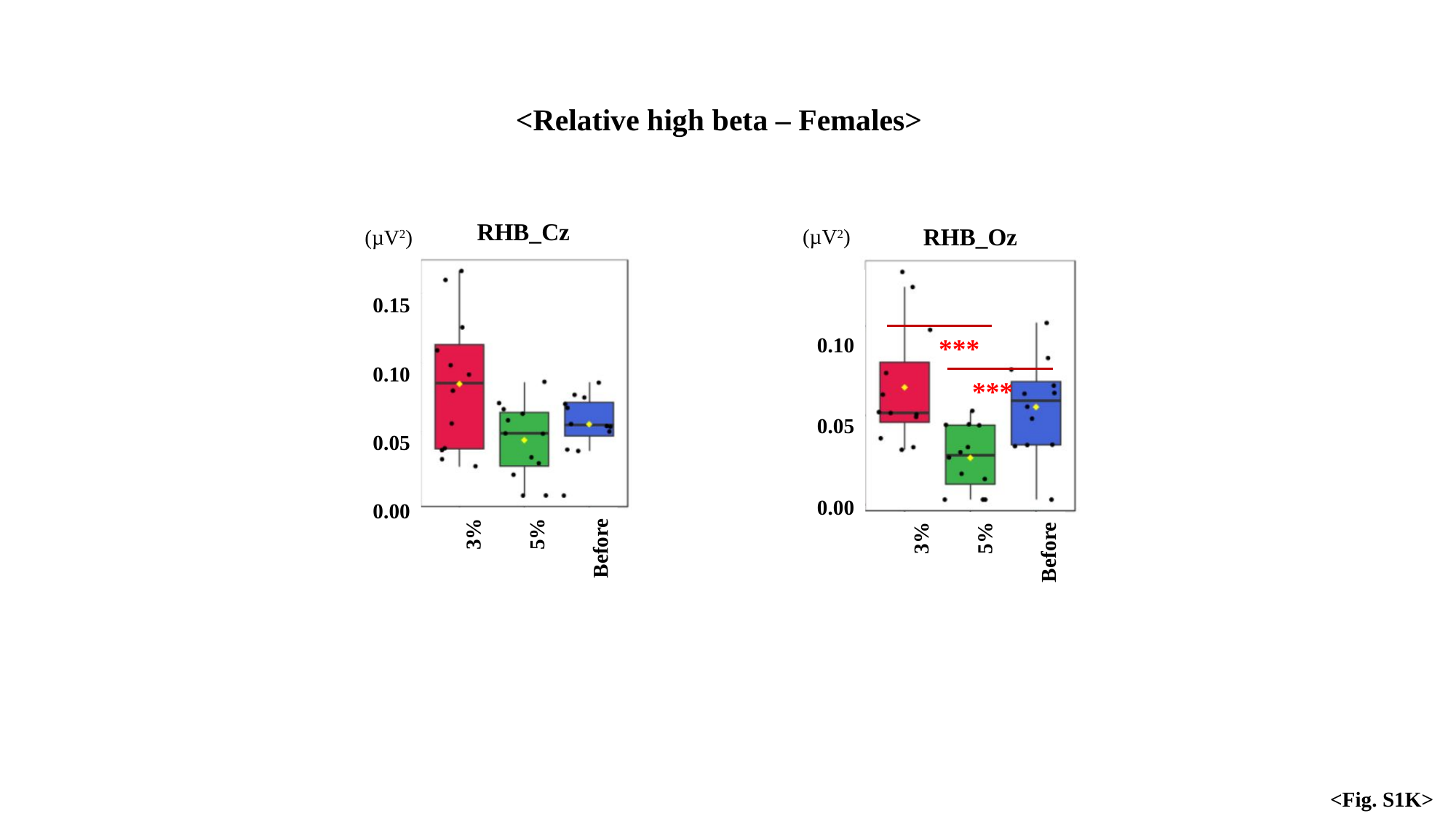

<Relative high beta – Females>
RHB_Cz
RHB_Oz
0.15
0.10
0.05
0.00
0.10
0.05
0.00
3%
5%
Before
3%
5%
Before
(µV2)
(µV2)
***
***
<Fig. S1K>
